# Freezing of gait and white matter changes: a tract-based spatial statistics study

**DOI:** 10.1186/s40734-014-0011-2

**Published:** 2015-01-20

**Authors:** Kazumi Iseki, Hidenao Fukuyama, Naoya Oishi, Hidekazu Tomimoto, Yoshinobu Otsuka, Manabu Nankaku, David Benninger, Mark Hallett, Takashi Hanakawa

**Affiliations:** Human Brain Research Center, Kyoto University Graduate School of Medicine, 54 Kawahara-cho, Shogoin, Sakyo-ku, Kyoto 606-8507 Japan; Human Motor Control Section, Medical Neurology Branch, National Institute of Neurological Disorders and Stroke, National Institutes of Health, Bethesda, MD USA; Department of Behavioral Neurology and Cognitive Neuroscience, Tohoku University, Graduate School of Medicine, Sendai, Miyagi Japan; Department of Neurology, Sakakibara-Hakuho Hospital, Tsu, Mie Japan; Department of Neurology, Mie University, Graduate School of Medicine, Tsu, Mie Japan; Department of Physical Therapy, Kyoto University Hospital, Kyoto, Japan; Department of Advanced Neuroimaging, Integrative Brain Imaging Center, National Center of Neurology and Psychiatry, Kodaira, Japan; PRESTO, JST, Kawaguchi, Saitama Japan

**Keywords:** Age-related white matter change (ARWMC), Diffusion tensor imaging (DTI), Disconnection, Freezing of gait (FOG), Tract-based spatial statistics (TBSS), Vascular Parkinsonism

## Abstract

**Background:**

We hypothesized that the integrity of white matter might be related to the severity of freezing of gait in age-related white matter changes.

**Methods:**

Twenty subjects exhibiting excessive hyperintensities in the periventricular and deep white matter were recruited. The subjects underwent the Freezing of Gait Questionnaire, computerized gait analyses, and diffusion tensor magnetic resonance imaging. Images of axial, radial and mean diffusivity, and fractional anisotropy were calculated as indices of white matter integrity and analyzed with tract-based spatial statistics.

**Results:**

The fractional anisotropy, mean, axial and radial diffusivity averaged across the whole white matter structure were all significantly correlated with Freezing of Gait Questionnaire scores. Regionally, a negative correlation between Freezing of Gait Questionnaire scores and fractional anisotropy was found in the left superior longitudinal fasciculus beneath the left premotor cortex, right corpus callosum, and left cerebral peduncle. The scores of the Freezing of Gait Questionnaire were positively correlated with mean diffusivity in the left corona radiata and right corpus callosum, and with both axial and radial diffusivity in the left corona radiata. The white matter integrity in these tracts (except the corpus callosum) showed no correlation with cognitive or other gait measures, supporting the specificity of those abnormalities to freezing of gait.

**Conclusion:**

Divergent pathological lesions involved neural circuits composed of the cerebral cortex, basal ganglia and brainstem, suggesting that freezing of gait has a multifactorial nature.

## Background

Freezing of gait (FOG) is a characteristic symptom observed in some patients with gait disorders. FOG is defined as an “inability to generate effective stepping movement” [1]. It is often observed in patients with idiopathic Parkinson’s disease (PD), and is even more prevalent in patients with atypical Parkinsonism (including vascular Parkinsonism) than in those with PD [2]. FOG can be seen in age-related white matter changes (ARWMC), which refer to the neuroradiological state of diffusely extended white matter lesions in elderly subjects [3-5].

The pathophysiology of FOG remains unclear [6]. The most reliable measure of the severity in FOG to date is the total score on the Freezing of Gait Questionnaire (FOGQ) [7]. Following recent development, white matter (WM) integrity can now be evaluated using diffusion tensor imaging (DTI). A directional bias of water diffusion can be quantified by measuring fractional anisotropy (FA), while mean diffusivity (MD) indicates the degree of diffusion [8].

Analysis of DTI can directly test whether the cortico-subcortical disconnection of WM tracts is responsible for gait disturbance as suggested by previous studies on gait disturbance in ARWMC [9,10]. Tract-based spatial statistics (TBSS) analysis of DTI data was recently developed as an automatic, hypothesis-free and precise method for the assessment of integrity of the WM [11]. Here we investigated the degree of integrity and directionality of the WM tracts in ARWMC patients, by means of TBSS analysis of DTI in combination with FOGQ scores.

## Methods

### Subjects

Twenty subjects with ARWMC were enrolled at the Neurology Clinic of Kyoto University Hospital. The inclusion criteria were: (1) aged between 65 and 84 years old, and (2) T_2_-weighted magnetic resonance images (MRI) revealing both irregular periventricular hyperintensities extending into the deep WM (Fazekas’ PVH 3) and confluent hyperintensities in the deep WM (Fazekas’ DWMH 3), diffusely involving at least bilateral fronto-subcortical areas and not confined to a single vascular territory [12]. Exclusion criteria were: (1) a history of acute stroke in the previous 3 months, (2) neurological and neuroradiological findings or history of surgery which suggests complication of other neurological or orthopedic diseases affecting gait including possible idiopathic normal pressure hydrocephalus (NPH). Specifically, an experienced neurologist first selected subjects solely according to the neuroimaging findings in the neurology clinic. The subjects whose diagnoses were other than ARWMC were excluded afterwards according to the exclusion criteria. The research protocol was approved by the ethics committee of Kyoto University Graduate School and Faculty of Medicine, and written informed consent was obtained from each subject.

### Assessment of FOGQ and gait performance

All subjects completed the FOGQ [7], and underwent neurological evaluation by a board-certified neurologist (K.I.). While subjects walked at their own pace repeatedly, the presence of FOG was checked, with special attention being paid to their gait initiation and turning. Moreover, they were assessed with a three-dimensional (3D) locomotion analysis system (GATAL-ITS-60, Sumitomo Metal Inc., Osaka, Japan). Velocity, cadence, stride length (SL), stride width (SW), gait cycle (GC), and the ratios of double support time per GC (DST/GC) were measured. The correlations between the gait-related parameters and the sum of FOGQ scores were tested (Spearman’s test). Additionally, the total score of FOGQ was compared between the FOG positive and FOG negative groups as classified by the clinical observation (Mann–Whitney *U* test). Findings were considered significant at the level of *P* <0.05.

### Assessment of cognitive status

Mini-mental state examination (MMSE) scores were obtained. They were not intended to provide a precise evaluation of cognitive impairment, as our main focus was the analysis of FOG. We conducted Spearman’s tests to test the correlations between FOGQ and MMSE scores. Findings were considered significant at the level of *P* <0.05.

### Image data acquisition

DTI, T_1_ and T_2_-weighted images were acquired with a 3-Tesla MRI scanner (Trio; Siemens, Erlangen, Germany). DTI data were obtained using a single-shot, spin-echo, echo-planar imaging sequence applying motion probing gradient pulses to 12 non-colinear axes with b = 700 s/mm^2^. To enhance the signal-to-noise ratio, imaging was repeated four times.

A magnetization-prepared rapid gradient echo (MPRAGE) sequence was used for anatomical T_1_-weighted volume data acquisition. T_2_-weighted images were acquired with a turbo spin-echo sequence.

### Data analysis

**Figure 1:**
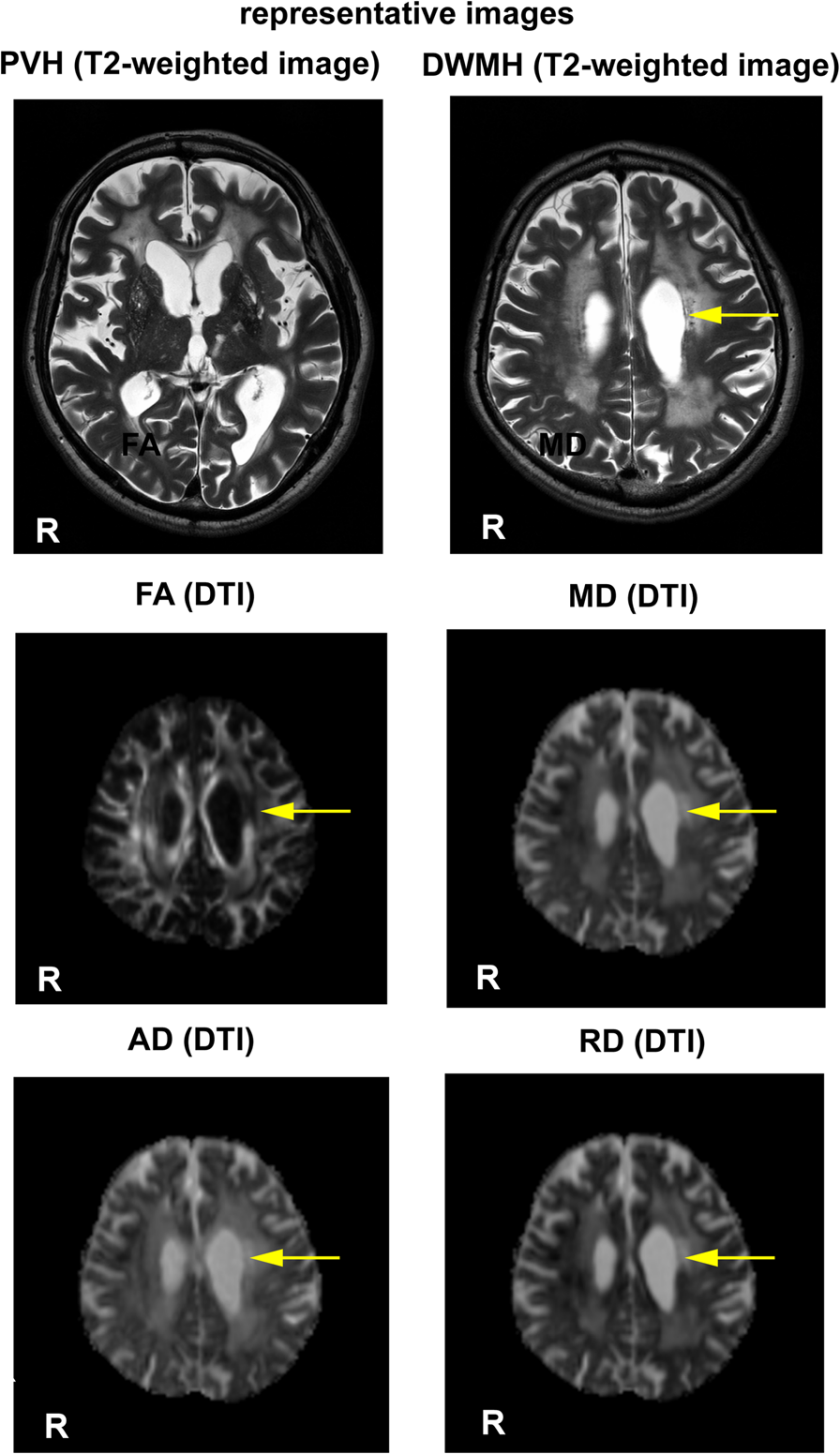
**Representative T**_**2**_ **-weighted images and maps of diffusion measures.** Representative T_2_-weighted images and maps of diffusion measures of a male subject who is 79 years of age are shown. The image at the upper left side represents irregular periventricular hyperintensities extending into the deep white matter (PVH), while the one at the upper right side represents confluent hyperintensities in the deep white matter (DWMH). Representative maps of FA, MD, AD and RD from the same subject are shown in the middle and lower side. The location shown in the diffusion maps is almost identical to the one demonstrated as DWMH. A part of the DWMH with T_2_-weighted image shows low FA, and high MD, AD and RD in the DTI as shown by the arrow colored with yellow.

The DTI data were pre-processed using DTI-Fit software included in the Functional MRI of the Brain (FMRIB) Diffusion Toolbox (FDT) (FSL4.1, http://www.fmrib.ox.ac.uk/fsl). We computed FA images and diffusivity images representing axial diffusivity (AD, λ_1_) and radial diffusivity (RD, λ_2_ + λ_3_/2) to the principal direction [13], and also mean diffusivity (MD). Representative T_2_-weighted images demonstrating PVH and DWMH, and maps of diffusion measures, both of which were taken from the same subject are shown in Figure 1.

First, we tested the relationship between the FOGQ score and the global changes of the diffusion measures reflecting different aspects of WM integrity (global correlation analysis). We hypothesized that FA would be decreased in accord with the severity of FOG (reflected by greater FOGQ scores) and that there would be a negative correlation between FOGQ and FA. As for the parameters representing diffusivity, MD is thought to reflect overall WM disruption similarly to FA, while RD and AD are thought to be useful as an index of myelin integrity and axonal integrity, respectively [13-15]. The most commonly reported pattern in previous studies is an increase in RD, AD, and MD in accord with the severity of age-related white matter damage [16]. We hence hypothesized that those diffusivity measures would be increased in relation to the severity of FOG and that there would be a positive correlation between FOGQ score and the three diffusivity parameters (MD, RD and AD). The correlation of FOGQ scores with each of FA, MD, AD and RD in the mean WM skeleton was tested using Spearman’s rank correlation.

To explore possible correlation between brain atrophy and FOGQ scores, the Structural Image Evaluation using Normalization of Atrophy (SIENAX) method [17] was then applied to the T_1_-weighted image for each subject to estimate the total, gray and white matter brain volumes.

Second, we tested regional correlations between the FOGQ score and the image representing each of the diffusion parameters (FA, MD, AD, and RD) on a voxel-by-voxel basis using the TBSS software. For the regional correlation analysis, we also speculated that the FOGQ score would be negatively correlated with FA values, and positively correlated with MD, AD and RD values. Statistical inference was obtained using a non-parametric method called Threshold-Free Cluster Enhancement (TFCE) with 5,000 permutations [18]. Changes were considered significant at the level of *P* <0.05, which was fully corrected for multiple comparisons. To test the specific relation of FOGQ scores with the diffusion measures identified in the regional correlation analysis, a confirmatory multiple linear regression analysis was applied. We computed the average value of FA, MD, AD and RD in each cluster, which showed significant regional correlation with FOGQ score as a sole dependent variable. The FOGQ scores, MMSE scores, walking velocity, cadence, SL, SW, GC and DST/GC were used as independent variables. Testing the multicollinearity that affects the result of the analysis is considered to be important when employing the multivariate analysis. Multicollinearity between these independent variables was tested by calculating the variance inflation factor (VIF), which is widely used for this purpose. If the VIF reveals specific variables to be unacceptable in the model, it is advised to exclude those specific variables. A well-accepted cutoff value of VIF is 10 [19,20]. The result was considered significant at the level of *P* <0.05.

## Results

### Behavioral parameters

Clinical assessment of the subjects, including the FOGQ scores and the presence of FOG, is shown in Table 1. The FOGQ scores (mean ± S.D.; 4.2 ± 5.0) were significantly correlated with the DST/GC (0.31 ± 0.074, *r* = 0.63, *P* = 0.003), walking velocity (0.98 ± 0.30, *r* = −0.79, *P* <0.001) and SL (0.96 ± 0.25, *r* = −0.74, *P* <0.001) (Table 2). Furthermore, there was a significant difference in FOGQ scores between subjects who were judged to exhibit FOG (9.2 ± 5.7, n = 6) and those who were not (2.5 ± 2.7, n = 14) (*P* = 0.003, Mann–Whitney *U* test). In addition, the FOGQ and the MMSE scores (28 ± 1.4) also showed a significant correlation (*r* = −0.62, *P* = 0.003; Table 2).

**Table 1:** **Clinical assessment of the subjects**

**Subject**	**Velocity (m/s)**	**SL (m)**	**DST/GC**	**FOGQ**	**Presence of FOG**
1	0.71	0.68	0.35	16	yes
2	0.58	0.8	0.33	6	yes
3	0.72	0.69	0.33	6	yes
4	0.54	0.59	0.46	17	yes
5	0.71	0.83	0.39	6	yes
6	0.62	1.2	0.46	4	yes
7	1.06	1.1	0.33	0	no
8	0.96	0.66	0.31	3	no
9	0.83	0.6	0.37	8	no
10	0.84	0.77	0.28	1	no
11	1.1	1.2	0.25	0	no
12	1.4	0.95	0.34	0	no
13	1.1	1	0.26	1	no
14	1.3	1.1	0.31	4	no
15	0.99	1.0	0.27	7	no
16	1.1	1.2	0.22	1	no
17	1.6	1.3	0.25	0	no
18	1.4	1.3	0.20	0	no
19	1.1	1.3	0.21	0	no
20	0.9	0.9	0.28	3	no

SL: stride length, SW: stride width, DST: double support time, GC: gait cycle, FOGQ: freezing of gait questionnaire, FOG: freezing of gait.

While subjects walked at their own pace repeatedly, the presence of FOG was checked, with special attention being paid to their gait initiation and turning.

**Table 2:** **The behavioral and DTI measures, and their correlations with FOGQ scores**

**Correlation with FOGQ scores**			
**Parameters**	**Mean ± S.D.**	***r***	***P***
DST/GC	0.31 ± 0.074	0.63	0.003*
Velocity	0.98 ± 0.30	−0.79	<0.001*
SL	0.96 ± 0.25	−0.74	<0.001*
MMSE score	28 ± 1.4	−0.62	0.003*
FA	0.38 ± 0.032	−0.49	0.026*
	(†0.45 ± 0.02)		
MD	0.99 ± 0.059	0.50	0.022*
	(†0.78 ± 0.03)		
AD	1.4 ± 0.042	0.48	0.029*
	(†1.18 ± 0.03)		
RD	0.79 ± 0.069	0.48	0.031*
	(†0.58 ± 0.04)		

For the DTI measures (FA, MD, AD and RD), values reported by Burzynska et al. using 63 healthy elderly subjects are shown in the parentheses as a reference. *P*-values considered to be significant are followed by an asterisk.

DST/GC: double support time/gait cycle, SL: stride length.

### Correlation of FOG with global changes in brain volumes and DTI measures

The assessment of brain atrophy with SIENAX failed to show a correlation between FOGQ scores and the volume of the total brain (*P* = 0.59), gray matter (*P* = 0.68) or white matter (*P* = 0.10). Nevertheless, when the diffusion measures averaged across the mean white matter skeleton were assessed, FOGQ scores showed a significant negative correlation with mean FA (0.38 ± 0.032 *r* = −0.49, *P* = 0.026), and a significant positive correlation with MD (0.99 ± 0.059 *r* = 0.50, *P* = 0.022), AD (1.4 ± 0.042, *r* = 0.48, *P* = 0.029) and RD (0.79 ± 0.069, *r* = 0.48, *P* = 0.031; Table 2).

### Local correlation of DTI measures with FOGQ scores

**Figure 2:**
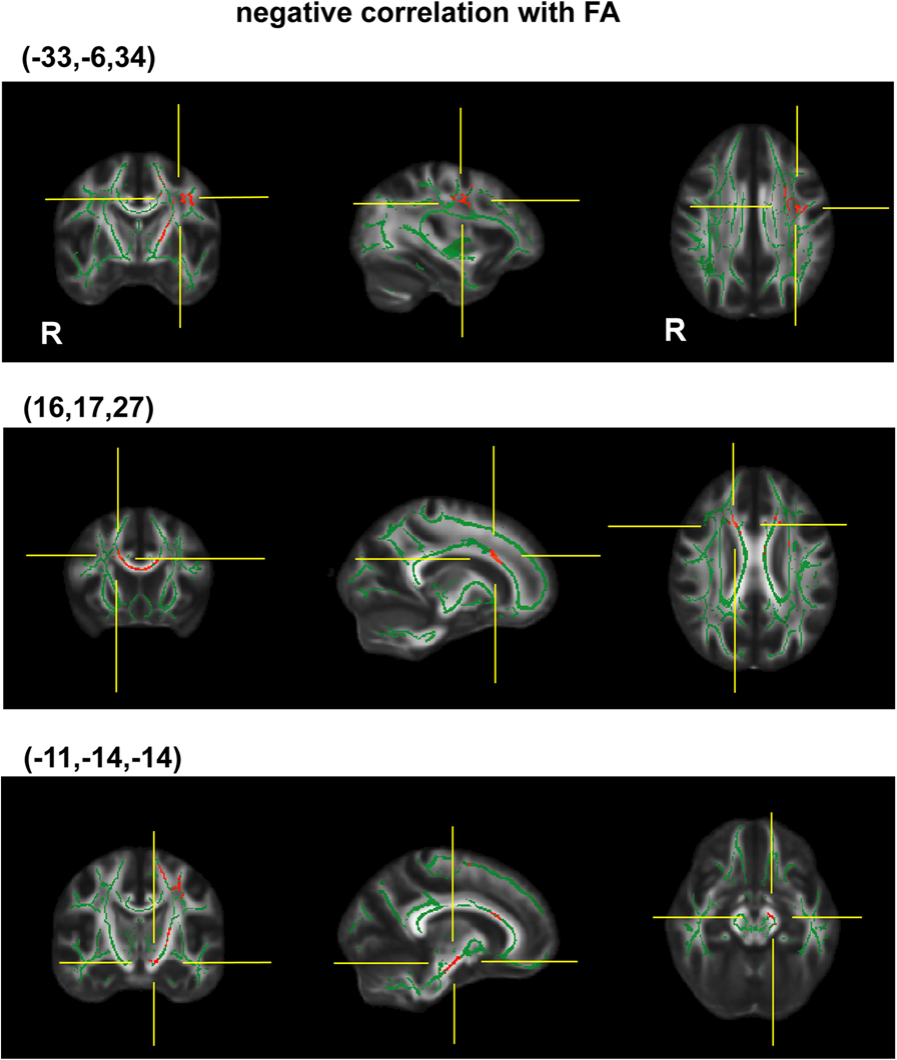
**Correlation between the FOGQ and FA.** The coordinates of detected regions within clusters that showed a significant correlation between the FOGQ and FA. The detected regions are colored with red and superimposed onto the white matter skeleton (green, thresholded at tract voxel FA >0.2) and the grayscale mean FA image of all the subjects. Note that the points of the crossed yellow lines represent the location with significant activation. See Tables 3 and 4 for details.

**Figure 3:**
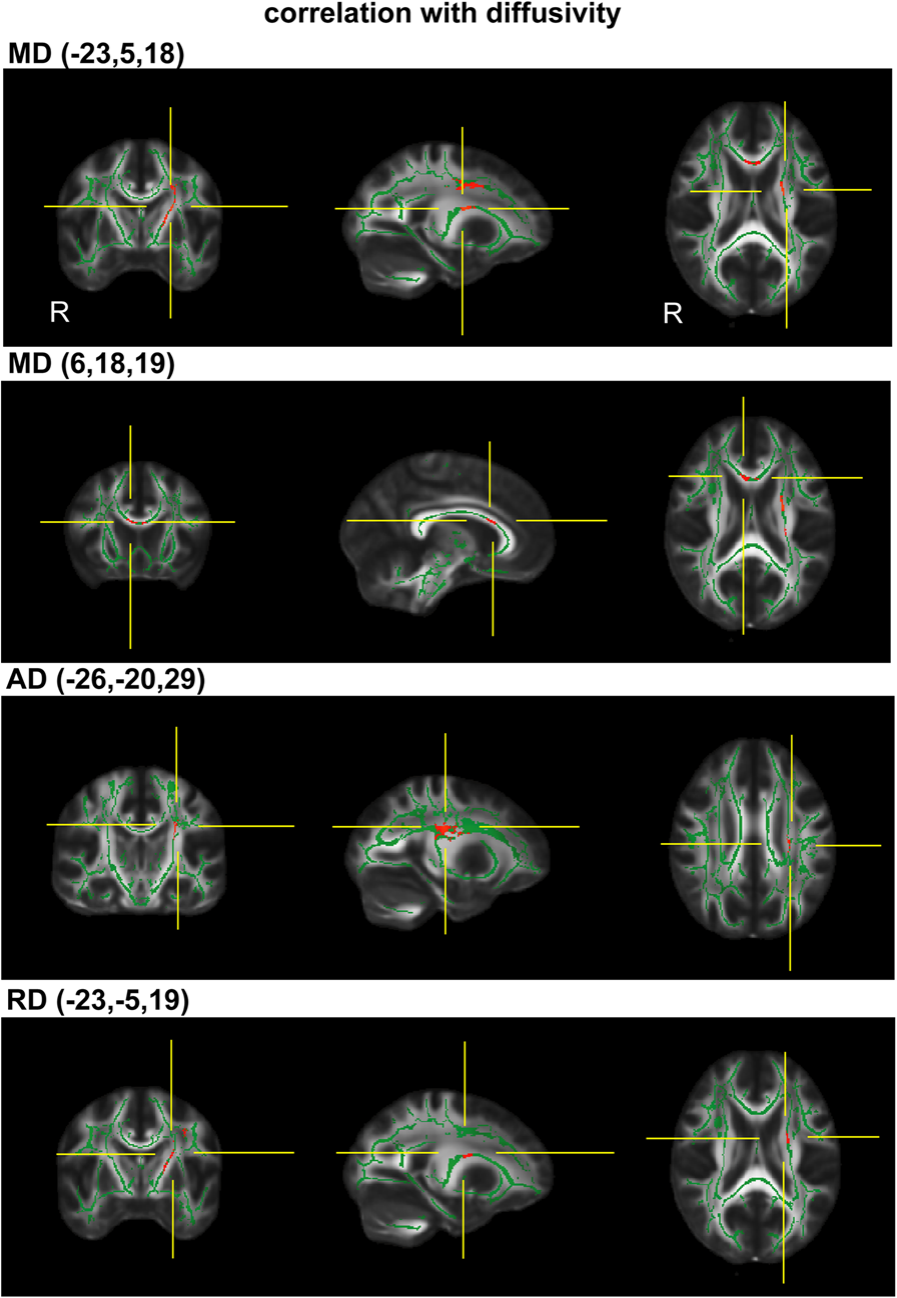
**Correlation between the FOGQ and diffusivity.** The detected regions showing significant correlations between the FOGQ and mean (A, B, MD), axial (C, AD) and radial diffusivity (D, RD). The detected regions are colored with red and rendered onto the template FA image. Note that the points of the crossed yellow lines represent the location with significant activation. See Table 3 and 4 for details.

In the TBSS analysis for the assessment of voxel-by-voxel local correlation, FA showed a significant negative correlation with the FOGQ scores in three clusters (Figure 2 and Table 3). They were composed of the left superior longitudinal fasciculus (SLF) located close to the premotor area (PM) (Brodmann’s area [BA] 6), the right corpus callosum (CC) nearby the anterior cingulate cortex (BA32), and the cerebral peduncle (CP) in the midbrain. A correlation analysis between the FOGQ scores and the MD revealed two significant WM clusters composed of the left posterior limb of the internal capsule (PLIC) located close to the putamen, and of the right cingulum close to the cingulate gyrus (BA24) (Figure 3 and Table 3). In addition, the FOGQ scores showed a significant positive correlation with the AD in the left superior corona radiata (SCR), and with the RD in the left SCR close to the putamen (Figure 3 and Table 3).

**Table 3:** **The location of each area which showed local correlation between DTI measures and FOGQ scores**

**Diffusion measure**	**MNI coordinates**			**Cluster size**	**Side**	**Area**	**Nearest GM**
	**x**	**y**	**z**				
FA	−33	−6	34	2060	left	SLF	precentral gyrus (BA6)
	16	17	27	732	right	CC	anterior cingulate (BA32)
	−11	−14	−14	196	left	CP	midbrain
MD	−23	−5	18	1037	left	PLIC	putamen
	6	18	19	178	right	cingulum	anterior cingulate (BA33)
AD	−26	−20	29	256	left	SCR	no GM found*
RD	−23	−5	19	173	left	SCR	putamen

SLF: superior longitudinal fasciculus, CC: corpus callosum, CP: cerebral peduncle, PLIC: posterior limb of the internal capsule, SCR: superior corona radiata, BA: Brodmann’s area, GM: gray matter.

*No gray matter was found around the 11×11×11-mm^3^ cube around this coordinate.

### Multiple linear regression analysis

To test how much of the variability in the regional diffusion measures was explained by FOG when other parameters were considered, multiple linear regression analyses were performed on the data extracted from the clusters detected by local correlation analysis. In the test of multicollinearity among the independent variables, VIF was less than 10 for the FOGQ score (2.8), MMSE score (3.7), SL (7.7), DSP (4.8), cadence (6.8) and SW (2.2) while it was more than 10 for the velocity (25) and GC (22). Since a well-accepted cutoff value of VIF is 10 [19,20], the walking velocity and GC were excluded from the independent variables. In this variable-reduced model, VIF was less than 3 for all the FOGQ (2.6), MMSE scores (1.8), SL (2.8), DSP (2.6), cadence (1.7) and SW (1.8). The adaptability of this reduced multi-regression model was significant for all dependent variables (*P* <0.05), supporting the appropriateness of the selected independent variables for predicting each dependent variable. In most of the WM regions, only the total FOGQ scores exhibited a significant association (*P* <0.05, Table 4). In the right CC, however, both the FOGQ score and the MMSE score exhibited significant associations.

**Table 4:** **Multilinear regression analysis**

**Diffusion measure**	**Area**	**Variables**	**Coefficient**	**T**	***P***
FA	left SLF	FOGQ	−0.81	−3.7	0.003*
		MMSE	0.43	2.0	0.067
		SL	−0.42	−1.8	0.092
		DST/GC	−0.30	−1.3	0.20
		Cad	−0.18	−0.99	0.34
		SW	0.14	0.76	0.46
FA	right CC	FOGQ	−0.57	−2.7	0.018*
		MMSE	0.60	3.4	0.005*
		SL	−0.27	−1.2	0.23
		DST/GC	0.02	0.093	0.93
		Cad	0.03	0.18	0.86
		SW	−0.18	−1.0	0.32
FA	left CP	FOGQ	−0.90	−4.28	<0.001*
		MMSE	0.45	2.0	0.071
		SL	−0.47	−2.2	0.063
		DST/GC	−0.08	−0.38	0.71
		Cad	−0.18	−1.1	0.30
		SW	−0.01	−0.06	0.95
MD	left PLIC	FOGQ	0.73	3.4	0.005*
		MMSE	−0.37	−2.1	0.07
		SL	0.15	0.64	0.53
		DST/GC	0.11	0.50	0.62
		Cad	0.16	0.92	0.38
		SW	0.03	0.18	0.86
MD	right cingulum	FOGQ	0.91	4.4	<0.001*
		MMSE	−0.26	−1.3	0.21
		SL	0.17	0.81	0.44
		DST/GC	0.11	0.55	0.59
		Cad	0.04	0.26	0.80
		SW	−0.37	−2.1	0.08
AD	left SCR	FOGQ	0.96	3.7	0.003*
		MMSE	0.05	0.22	0.83
		SL	0.21	0.76	0.46
		DST/GC	−0.17	−0.63	0.54
		Cad	−0.06	−0.3	0.77
		SW	0.14	0.65	0.53
RD	left SCR	FOGQ	0.69	2.7	0.019*
		MMSE	−0.08	−0.39	0.71
		SL	−0.06	−0.21	0.84
		DST/GC	0.12	0.48	0.64
		Cad	0.02	0.11	0.91
		SW	−0.07	−0.32	0.75

The table shows the results of multilinear regression analysis of all those six independent variables using average activation of each area in which significant local correlation between FOGQ and FA was found as a dependent variable. The significant *P*-values are noted by an asterisk.

FOGQ: Freezing of Gait Questionnaire, MMSE: Mini-Mental State Examination, SL: stride length, DST/GC: double support time/gait cycle, cad: cadence, SW: stride width.

Further, to test the effects of the velocity and GC that were excluded from the reduced multi-regression model, we replaced the FOGQ with the velocity or GC in another series of multiple regression analysis. When the velocity was used, the adaptability of the model was found to be non-significant in all WM clusters, except the right CC (*F* = 4.06, *P* = 0.016). In the right CC, only MMSE scores significantly explained the variance in the diffusion-derived variables (*P* = 0.045). When the GC was substituted for the FOGQ scores, the adaptability of the model was non-significant in all WM clusters, except for the right CC (*F* = 4.05, *P* = 0.016). Again in the right CC, only the MMSE scores revealed significant effects (*P* = 0.012).

This analysis suggested that DTI measures of the WM regions, except for the right CC, were specifically correlated with the FOGQ scores, but not with other cognitive or gait parameters.

## Discussion

### Assessment of FOG

The present study revealed a strong correlation between the FOGQ scores and the gait parameters from the computerized gait analysis. PD subjects with FOG have gait disturbance, which result in the abnormality of the gait parameters. For example, FOG affects GC which results in the increase of the stride-to-stride variability [21]. Furthermore, gradual reduction of stride length is associated with FOG [22]. The result from the present study concurs with these findings, although we should be careful not to simply equate FOG and gait disturbance. That is, although most subjects with FOG show profound gait disturbance, some subjects with FOG may show normal gait parameters upon a gait analysis, reflecting fluctuating nature of FOG [6].

Nevertheless, the multiple regression analysis indicated that only the FOGQ score exhibited a significant effect as an independent variable explaining changes of the DTI measures in almost all the WM clusters detected in the local correlation analysis. The multiple regression analysis substituting the FOGQ scores for the walking velocity or GC failed to produce significant results in the model in most of the WM clusters. These results indicate that the diffusion measures in the WM clusters (except for CC) were strongly correlated with the severity of FOG specifically, not with cognitive decline or gait disturbance in general.

### Interhemispheric connections

In the CC, FA exhibited a significant negative correlation with FOGQ and MMSE scores. WM integrity of the CC is significantly correlated with quantitative measures of gait in the elderly population [9]. It has been suggested that the CC may transfer sensory information necessary for gait planning interhemispherically [23]. Moreover, a recent study demonstrated that perceptual judgment of an upcoming doorway is more strongly affected in patients with FOG than those without [24]. These findings suggest that CC may affect the ability of gait planning according to sensory information in the environment which might be related to FOG.

### Corticofugal and thalamocortical fibers

The present study revealed a negative correlation between FOGQ scores and FA in the ventromedial part of the CP at the midbrain level. The CP includes the corticofugal tract, which connects the brainstem and the spinal cord with cortical motor areas including the primary motor cortex, supplementary motor area (SMA) and PM [25]. The CP may also include the fibers projecting to the pedunculopontine nucleus (PPN) and/or the mesencephalic locomotor region (MLR). The neural circuits connecting the PPN, basal ganglia, motor cortex and limbic system are hypothesized to be responsible for FOG [26]. Moreover, recent study revealed that the PD patients with FOG showed more increase of brain activity in the PPN/MLR during imagery of walking compared with those without FOG [27]. Taken together, the disruption of the projecting fibers to PPN/MLR at the level of CP appears to be responsible for FOG.

There was a positive correlation between MD in the PLIC and FOGQ scores. Damage to the PLIC encompassing parts of the corticofugal tracts could result in poor motor outcomes [28]. In infants with low birth weight, reduced FA in the PLIC has been shown to be correlated with the severity of gait deficits in the future [29].

Both AD and RD were significantly correlated with the FOGQ scores in the left SCR. Hence, the damage to both myelin and axons might contribute to the WM damage seen here. Damage of the thalamocortical sensory tracts at the SCR can cause both sensory deficits and gait disturbance [30]. Considering that FOG can be ameliorated or exacerbated by sensory stimuli [31], sensorimotor networks are likely to be involved.

### Fronto-parietal connections and cingulum

It has been suggested that FOG may result from dysfunction of the PM-parietal circuits [32]. Consistent with this view, we found a significant negative correlation between FA and FOGQ scores in the SLF close to the PM. The SLF is likely to contribute to visuospatial processing [33] which might be related to FOG.

The cingulum collects projections from the cingulate cortex and extends into the temporal or frontal lobe [34]. In the present study, MD in the cingulum exhibited a positive correlation with FOGQ, consistent with the hypothesis that a local network in SMA and adjacent cingulate cortex might be responsible for FOG [35]. The cingulate regions are activated during gait movement [10,36] and are also thought to be involved in the planning of gait [37,38]. The cingulum bundle participates in the limbic circuit, which is thought to be involved in FOG together with the motor and cognitive circuits in patients with PD [26]. Based on these considerations, the present findings in the cingulum might reflect not only the motor but also the emotional aspects of FOG.

### Brain volume, global diffusion measures and FOG

Previous studies indicate that gray matter atrophy is related to gait impairment [39], as well as the volume of WM lesions [40]. However, our analysis failed to reveal a significant correlation between WM atrophy and FOGQ, whereas all of the diffusion measures were significantly correlated with FOGQ. Thus, it appears that the diffusion measures were more sensitive than WM atrophy in patients with ARWMC.

We did not take the WM lesion volume into account in the TBSS analysis since we focused on the integrity of the white matter. However, we could not exclude a possibility that such WM volume changes might affect the TBSS results.

### Proposed mechanisms underlying FOG

The present findings raise the question of how the WM abnormalities identified above (CC, PLIC, SCR, CP, and SLF) might explain FOG. Youn et al. [41] performed a region-of-interest (ROI) analysis on DTI data and found that the FA values of the bilateral PPN, bilateral PM, right orbitofrontal area and left SMA were lower in the ARWMC patients with FOG compared to those without. Their finding partly concurs the present result, which showed that the FA in the SLF close to the PM, and also in the CP close to the midbrain PPN, was correlated with FOG. The areas detected slightly differed probably because of the difference in the methodology, especially the spatial normalization procedure (TBSS versus ROI analysis). The present result suggests that multiple neural substrates related to the control of walking may be involved in FOG in consistent with the study by Youn et al. [41]. This notion favors the view that FOG is likely to be a clinical condition resulting from multiple pathophysiological issues involving one or more nodes of the neural network regulating walking behavior. Since visual stimuli or some other extra cues can help or exacerbate FOG [31], the phenomenon might emerge when extra demands for sensory, cognitive, or emotional processing overburden the damaged neural substrates.

### Limitations of this study

We enrolled patients who were regularly visiting a neurology outpatient clinic. Thus, the population might be different from those who were diagnosed as having ARWMC in radiology departments or in general elderly population. Therefore, a further study will be required to test if the present finding can be generalized to those population.

In the present study, we focused on the relationship between WM abnormality and FOG. We employed a relatively small number of subjects to perform a comprehensive investigation. Since there is no established objective measure of FOG, we avoided classifying subjects with FOG and those without, and used FOGQ as a parameter for a correlation analysis instead. Because of this strategy, we have included the subjects who did not clearly present FOG, which is often invisible at the physician’s room. To reduce ambiguity regarding the subject selection, we did our best to discriminate FOG and general gait disturbance in the multiple regression analysis, but it would still be difficult to discuss the mechanisms of FOG completely segregated from those of gait disturbance in general.

In the present study, we used the T_2_-weighted images only for the purpose of subject recruitment, but did not analyze these non-quantitative data. We reviewed the individual T_2_-weighted images of each subject, and visually investigated if the regions detected in the DTI analysis corresponded to ARWMC regions in the T_2_-weighted images. In most of the subjects, ARWMC were present in all the detected regions except for the CP. This result is reasonable since we tested the correlation between the FOGQ scores and the diffusion parameters, which should reflect different aspects of anatomical structure seen in the T_2_-weighted images. For example, as minor reduction of white matter integrity resulting from remote ischemic lesions would be captured by DTI, but not necessarily by T_2_-weighted images. Future research comparing DTI and quantitative T_2_-weighted images is awaited. We discussed FOG mainly in relation to the underlying pathophysiology of FOG in PD. The symptoms of FOG seem very similar across different diseases such as PD and other types of Parkinsonism. However, it remains unclear whether the mechanisms underlying FOG are shared across related diseases. Therefore, caution must be exercised in generalizing the mechanisms of FOG suggested here. Future studies are necessary to compare the mechanisms of FOG across different diseases.

## Conclusion

While disconnections in the brain have previously been suggested as a cause of FOG, few studies have directly addressed this issue. To our knowledge, the present study provides the first experimental evidence that FOG is related to WM disruption, with the current results revealing that WM damage in the SLF, CC, PLIC, cingulum, CR and CP was correlated with FOG.

## Abbreviations

ARWMC: Age related white matter change
FA: Fractional anisotropy
FOG: Freezing of gait
PD: Parkinson’s disease
